# Responses of Anti-Apoptotic Molecules to EAgNPs and EAgNPs Plus L-Buthionine-Sulfoximine in Human Tongue Cancer Cells

**DOI:** 10.3390/molecules31193536

**Published:** 2026-10-04

**Authors:** Ximing Wu, Fuming Wang, Xiaoyu Huang, Mingchuan Yang, Yufeng He, Jinsong Zhang, Huali Wang

**Affiliations:** 1 Anhui Province Green Food Collaborative Technology Service Center for Rural Revitalization, School of Biological and Food Engineering, Hefei Normal University, Hefei 230601, China; wxm2022@hfnu.edu.cn; 2 State Key Laboratory of Tea Plant Biology and Utilization, School of Tea & Food Science, Institute of Health and Medicine, Hefei Comprehensive National Science Center, Anhui Agricultural University, Hefei 230601, Chinaqhyf2019@ahau.edu.cn (Y.H.); 3 Department of Medical Devices, Shandong Drug and Food Vocational College, Weihai 264210, China; yangmingchuan@tricaas.com; 4 Tea Research Institute, Chinese Academy of Agricultural Sciences, Hangzhou 310008, China

**Keywords:** silver nanoparticles, human tongue cancer cells, selenoprotein, drug resistance, glutathione, Nrf2-associated antioxidant proteins

## Abstract

Silver nanoparticles (AgNPs) have emerged as a promising nanoplatform for oncological intervention, yet their clinical translation is severely hampered by systemic toxicities associated with high effective doses, while the underlying mechanisms governing their interactions with cancer cell pro-survival cascades remain poorly defined. In this study, we synthesized and fully characterized green tea polyphenol (-)-Epigallocatechin-3-gallate (EGCG)-derived silver nanoparticles (EAgNPs), in which EGCG acts as the reducing and capping agent. EAgNPs were confirmed to be highly dispersed, stable metallic silver particles with a primary size range of 4–7.6 nm. Subsequent mechanistic investigations performed on Tca8113 human tongue cancer cells revealed distinct dose-dependent anticancer action modes of EAgNPs: the 100 μM lethal dose effectively triggered robust apoptosis via potently suppressing the activity of key antioxidant selenoenzymes, including thioredoxin reductase and glutathione peroxidase (GPx), and multiple drug resistance proteins. Notably, even under this lethal treatment condition, Tca8113 cells still initiated theNrf2-associated antioxidant response characterized by 2-fold glutathione elevation and 25-fold heme oxygenase-1 upregulation. The 30 μM low-dose EAgNPs, however, failed to produce salient cytotoxicity, as they moderately inhibited GPx activity while comprehensively activating the integrated cancer cell defense network, including glutathione accumulation, Nrf2-associated antioxidant protein induction, and upregulation of a full panel of drug resistance proteins. Interestingly, the combination of 30 μM EAgNPs with the glutathione synthesis inhibitor L-buthionine-sulfoximine further enhanced selenoenzyme GPx inhibition and completely abrogated all above-mentioned inducible anti-apoptotic responses, resulting in multi-target disruption of the cytoprotective system and strongly enhanced apoptosis induction. Our findings demonstrate that this combinatorial strategy effectively lowers the therapeutic dose threshold of EAgNPs by disabling cancer cell adaptive resistance, providing a low-toxicity, translationally feasible approach for localized oral cancer treatment.

## 1. Introduction

Extensive in vitro and in vivo investigations consistently demonstrate that silver nanoparticles (AgNPs) exhibit potent cytotoxic effects against a wide spectrum of malignant cell types [1,2,3,4]. Following cellular internalization via endocytosis, AgNPs dissociate to release Ag+ ions, which trigger a robust burst of intracellular reactive oxygen species (ROS) [5,6,7,8,9,10]. This oxidative surge subsequently induces DNA damage, driving dose-dependent apoptotic death in cancer cells. Despite this well-documented tumoricidal potential, the clinical translation of AgNPs continues to face substantial unaddressed barriers, and the exact molecular mechanisms governing their interactions with critical cancer cell pro-survival cascades remain largely unclarified.

Selenoenzymes, the core regulatory components of cellular redox homeostasis [11], are widely expressed across both normal and tumor tissues [12], performing indispensable functions in eliminating excess ROS, sustaining intracellular redox equilibrium, and shielding cells from oxidative damage [13,14]. To counteract the persistent oxidative stress brought on by rapid proliferation and hyperactive metabolic activity, malignant cells frequently upregulate multiple selenoenzymes as a key survival strategy [15,16,17]. Thioredoxin reductase (TXNRD), one of the most pivotal selenoenzymes, supports cancer cell adaptation to metabolic and replication-induced redox stress [17].

Targeted disruption of this cancer cell-specific antioxidant defense network to amplify oxidative damage is thus recognized as a viable and promising anti-tumor therapeutic strategy. TXNRD exhibits uniquely high susceptibility to silver species, and previous toxicological research has verified that AgNPs and dissociated Ag^+^ ions can inhibit selenium incorporation into selenoenzymes in a dose-dependent manner, drastically reducing both the expression and enzymatic activity of TXNRD [18].

This inhibitory effect is identified as the central driver of silver-induced oxidative stress and subsequent cell growth arrest [18]. However, tumor cells can launch adaptive protective responses to resist AgNPs-triggered oxidative injury: the suppression of TXNRD often initiates compensatory activation, including the Nrf2-associated antioxidant proteins, leading to the upregulation of downstream antioxidant mediators such as heme oxygenase-1 (HO-1) and NAD (P)H quinone oxidoreductase 1 (NQO1) [19,20,21]. The cumulative antioxidant effect of these molecules alleviates oxidative damage initiated by AgNPs, thus reducing the tumoricidal potential of AgNPs [22].

In addition, cancer drug resistance by multidrug resistance proteins [23] and glutathione S-transferase (GST) [24] remains a primary obstacle limiting the efficacy of clinical chemotherapy [25]. Drug resistance allows cancer cells to evade the cytotoxic effects of anti-tumor agents, ultimately resulting in treatment failure, tumor recurrence, and disease progression [26,27]. Given the critical roles of TXNRD, GSH, and theNrf2-associated antioxidant proteins in tumor oxidative stress adaptation, this study puts forward a key unresolved scientific hypothesis: i) during AgNPs-mediated anti-tumor therapy, TXNRD inhibition by AgNPs may induce GSH and Nrf2-associated adaptive cytoprotection that counteracts the cytotoxic effects of AgNPs and promotes the development of intrinsic drug resistance in cancer cells, and (ii) a compound that could simultaneously suppress all these adaptive responses would greatly synergize with AgNPs to kill cancer cells.

Green tea’s predominant bioactive catechin, (−)-Epigallocatechin-3-gallate (EGCG) has been widely adopted as an efficient, environmentally friendly reducing and capping agent for the biosynthesis of silver, gold, and ruthenium nanoparticles [28,29,30,31]. Compared with conventional chemical synthesis methods, EGCG-mediated biosynthesis features simple operational procedures and endows the nanoparticles with excellent dispersion stability and biological compatibility [32,33,34]. In the present research, we successfully biosynthesized EGCG-derived silver nanoparticles (EAgNPs), and used human Tca8113 tongue cancer cells as the experimental model to investigate the anti-tumor and pro-apoptotic mechanisms of EAgNPs, with a specific focus on exploring the regulatory effects of EAgNPs on selenoenzyme activity, intracellular GSH levels, the Nrf2-associated antioxidant proteins, and tumor drug resistance proteins. In addition, we evaluated cancer cell adaptive resistance against EAgNPs plus the glutathione synthesis inhibitor L-buthionine-sulfoximine (BSO). We found that BSO abrogated all examined compensatory protective responses induced by EAgNPs; therefore, the combination of EAgNPs and BSO produced enhanced pro-apoptotic activity.

## 2. Materials and Methods

### 2.1. Reagents

EGCG (99%), purified from green tea, was obtained from Ebeikar Tea & Extracts Co., Ltd. (Hangzhou, China). AgNO_3_ was purchased from Shanghai Shenbo Chemical Co., Ltd. (Shanghai, China). L-Buthionine-sulfoximine (L-BSO) and the primary antibody against β-actin were purchased from Sigma (St. Louis, MO, USA). 3-(4,5-Dimethyl-2-thiazolyl)-2,5-diphenyltetrazolium bromide (MTT) was purchased from Amresco LLC (Solon, OH, USA). The primary antibodies as well as the anti-mouse and anti-rabbit secondary antibodies were purchased from Cell Signaling Technology (Boston, MA, USA). Fetal bovine serum was purchased from BioExcel (Shanghai, China). RPMI 1640 medium, trypsin, and penicillin-streptomycin solutions were purchased from HyClone (Logan, UT, USA). Other chemicals were of the highest grade available.

### 2.2. Cell Culture

Tca8113 human tongue cancer cell line was provided by the Key Laboratory of Oral Biomedicine of Shanghai Jiaotong University (Shanghai, China). Cancer cells were cultured in RPMI 1640 medium containing 10% (*v*/*v*) fetal bovine serum, 100 μg/mL streptomycin, and 100 U/mL penicillin. All cells were cultured under 5% CO_2_ at 37 °C.

### 2.3. Preparation of EAgNPs

EGCG is capable of serving as an effective reducing and stabilizing agent for nanoparticle fabrication [32,33,34]. The preparation method of EAgNPs was to add 1 mM EGCG in water at pH 11.5 and then added 1 mM AgNO_3_ in vortex drops. After 5 min, a stable red-brown nano-silver solution was obtained with pH 7.0–8.0. No additional purification step was performed for the as-prepared EAgNPs reaction mixture, and the original reaction dispersion was directly used for subsequent cell experiments. The concentration of EAgNPs reported in cell experiments was calculated based on the initial molar concentration of AgNO_3_ adopted in the synthesis. EAgNPs stock solution was preserved as an aqueous dispersion and not dried before use. For cell treatment, the stock dispersion was directly added into complete cell culture medium to prepare target working concentrations.

### 2.4. Characterization of EAgNPs

#### 2.4.1. UV—Visible Spectrum Analysis

The UV—visible absorption spectrum of the EAgNPs aqueous dispersion was recorded using a UV-1800 spectrophotometer (SHIMADZU, Kyoto, Japan). The scanning wavelength range was set from 200 nm to 600 nm, and deionized water was used as the blank reference for baseline correction before sample measurement.

#### 2.4.2. Dynamic Light Scattering (DLS) Measurement

The hydrodynamic particle size and zeta potential of EAgNPs were determined by DLS (DelsaMax PRO, Beckman, Krefeld, Germany). The freshly prepared 1 mM EAgNPs aqueous dispersion was diluted 10-fold using pure water. Then 2 mL of diluted samples were subjected to and transferred into a disposable polystyrene cuvette for detection. The instrument was equipped with a 633 nm red laser, and the detection angle was set at 173° (backscatter mode). All measurements were performed at 25 °C. Each sample was tested three times and each acquisition lasted 10 s. The average hydrodynamic diameter and zeta potential were recorded, and the polydispersity index (PDI) was automatically calculated by the system software.

#### 2.4.3. Transmission Electron Microscopy (TEM) Analysis

The microstructure, morphology, and particle size distribution of EAgNPs were observed by TEM (HT7700, Hitachi, Tokyo, Japan). For sample preparation, 10 μL of 10-fold diluted EAgNPs aqueous suspension was dropped onto a carbon-coated copper grid (200 mesh) and naturally dried at room temperature. TEM imaging was performed at a working accelerating voltage of 200 kV. Multiple fields of view at different magnifications (ranging from 5000× to 100,000×) were randomly captured to characterize nanoparticle morphology, dispersion state, and particle size. The diameter of the particles was determined using ImageJ software(version 1.54f), ensuring that a minimum of 100 nanoparticles were counted to derive the average size and distribution.

#### 2.4.4. Fourier Transform Infrared (FT-IR) Spectroscopy Analysis 

The functional groups of EAgNPs were characterized byFT-IR (Nicolet IS10, Thermo, USA). Before detection, the freshly prepared EAgNPs aqueous dispersion was frozen and vacuum freeze-dried to obtain solid nanoparticle powder. The dried EAgNPs powder was uniformly ground with dry KBr powder at a mass ratio of approximately 1:100 and pressed into a transparent tablet for testing. The scanning range was set from 4000 cm^−1^ to 500 cm^−1^, with a resolution of 4 cm^−1^ and a total of 32 cumulative scans per spectrum. Background scanning was performed and automatically deducted before sample measurement to eliminate interference from water vapor and carbon dioxide in air.

#### 2.4.5. X-Ray Diffraction (XRD) Characterization

XRD analysis was performed on a Bruker D8 Advance diffractometer with Cu Kα radiation (λ = 0.15406 nm) operated at 40 kV and 40 mA. Samples were scanned over a 2θ range from 10° to 80° at a scanning rate of 2°/min. The obtained diffraction patterns were matched against standard PDF reference cards to identify crystal phases.

#### 2.4.6. X-Ray Photoelectron Spectroscopy (XPS) Characterization

XPS was performed using a Thermo Scientific ESCALAB 250Xi spectrometer (Thermo Scientific, Waltham, MA, USA), utilizing Al Kα radiation (1486.6 eV) for excitation. The system operated under a pressure of less than 10^−9^ mbar. Calibration of the acquired spectra was achieved through the C 1s peak at 284.8 eV. The binding energy peaks corresponding to Ag 3d were examined to determine the chemical state of sliver within EAgNPs.

### 2.5. HPLC Assay

EGCG content was detected by high-performance liquid chromatography (HPLC) (Waters Instruments, Inc., Rochester, MN, USA) according to our previously reported method [35]. Briefly, chromatographic separation was performed on a Symmetry^®^ C18 (Waters, Milford, MA, USA), 5 μm, 110 A column, 250 × 4.6 mm. The mobile phase consisted of (A) deionized water with 0.15% acetic acid and (B) acetonitrile. The following gradient was applied: 0 min at 85% A, a linear gradient to 75% A for 13 min, then a linear gradient to 10% A for 1 min, held for 4 min, and returned to 85% A for 1 min, then maintained for 6 min to balance the system. The column temperature was set at 30 °C. The injection volume of samples was 10 µL. The elution rate was 1 mL/min, and the detection wavelength was set at 278 nm.

### 2.6. MTT Assay

Tca8113 cells were plated overnight at 10,000 cells/well in 96-well plates. Cells were then treated with the indicated agents for 24 h. After that, the MTT assay was done as previously described [36].

### 2.7. Cell Migration

Tca8113 cells were plated overnight at 1 × 10^6^/well on 6-well plates. Sterile pipette tips were used to scratch cell layers. The cells were then washed with phosphate-buffered saline (PBS) and treated with EAgNPs for 48 h and photographed at the indicated times. The relative migration ratio was calculated according to the formula: (width at 0 h—width at 48 h)/width at 0 h.

### 2.8. Colony Formation

Tca8113 cells were plated overnight in 6 cm cell culture dishes at 500 cells/dish and treated with the EAgNPs for 10 days. Following a brief wash with PBS, the cells were fixed with methanol for 15 min and stained with 0.1% crystal violet for 20 min to count colony numbers.

### 2.9. GSH Assay

GSH content was measured according to our previously reported method [37]. Briefly, cells were homogenized with saline and mixed with 20% (*w*/*v*) trichloroacetic acid to precipitate protein at a ratio of 9:1 (*v*/*v*) and then centrifuged at 10,000 *g* at 4 °C for 15 min. The precipitate was discarded and the supernatant was mixed with DTNB and read at 412 nm. Data were presented as nmol/mg protein.

### 2.10. Measurement of TXNRD Activity

We utilized NADPH-dependent DTNB reduction that is inhibited by auranofin as a measure of TXNRD activity, which was analyzed essentially according to the method of Smith and Levander [38] with some modifications, shortly summarized as follows. First, a stock mixture was freshly prepared and kept at 37 °C. For analyses, cell homogenate was mixed with auranofin solution or 5% ethanol at a ratio of 9:1 (*v*/*v*), at 37 °C for 10 min. After adding 50 μL of both paired samples to a 96-well plate, the reactions were started by the addition of 250 μL of the stock mixture to each sample. Changes in absorbance at 412 nm over time were monitored for the first 3 min at 37 °C using a microplate reader. The cytoplasmic TXNRD activity was expressed as U/mL.

### 2.11. Measurement of GPx Activity

Glutathione peroxidase (GPx) was measured following the method of Smith and Levander [38] with 300 μM of H_2_O_2_ as substrate, coupled with GR and NADPH to reduce oxidized glutathione. GPx activity was calculated in terms of μmols of NADPH oxidized/min/mL cytoplasm (U/mL).

### 2.12. Western Blot

Total cell proteins were extracted and separated by SDS-PAGE and transferred to a polyvinylidene difluoride membrane. Western blot analysis was performed as described previously [36] using the specific primary and secondary antibodies. Proteins were detected using the ChemiDoc XRS+ detection system (ECL; Bio-Rad, Hercules, CA, USA). The Quantity One Image Analyzer software program (Bio-Rad, Hercules, CA, USA, version 4.6.9) was used for quantitative densitometric analysis.

### 2.13. Statistical Analysis

Data are presented as means ± SEM. The differences between groups were examined by Student’s *t*-test or one-way ANOVA post hoc Dunnett test, as appropriate. All statistical analyses were performed using GraphPad software (Prism version 5, San Diego, CA, USA). A *p*-value of < 0.05 was considered statistically significant.

## 3. Results

### 3.1. Preparation and Characterization of EAgNPs

**Figure 1:**
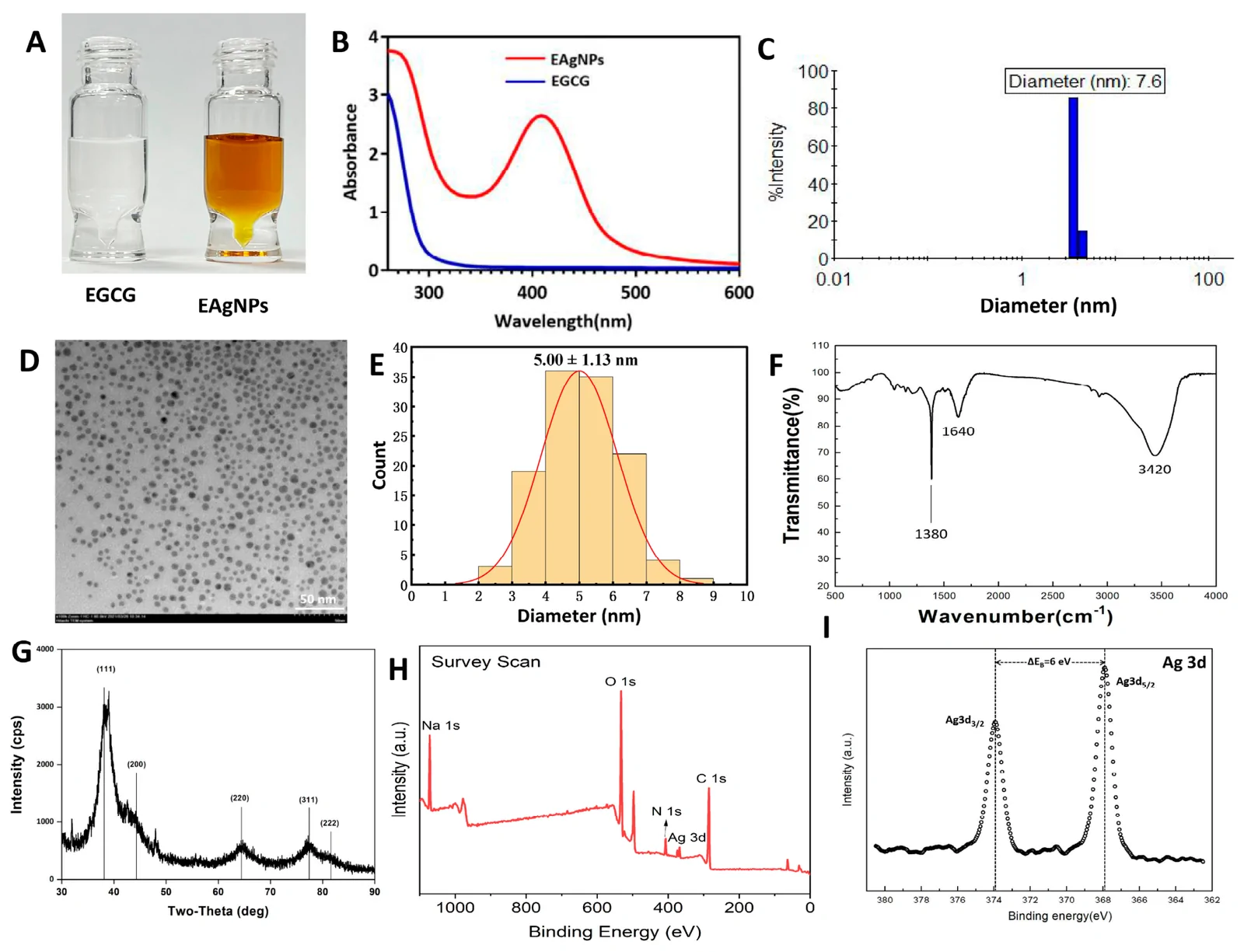
Preparation and characterization of EAgNPs. (**A**) Photographs of EGCG solution and the as-prepared EAgNPs dispersion. (**B**) Ultraviolet-visible absorption spectra of EAgNPs. (**C**) Hydrodynamic size distribution of EAgNPs determined by DLS. (**D**) TEM image of EAgNPs. (**E**) Histogram of particle size distribution generated from TEM images. (**F**) FT-IR spectrum of EAgNPs. (**G**) XRD spectrum of EAgNPs. (**H**) XPS survey scan of EAgNPs. (**I**) High-resolution XPS spectra of Ag 3d in EAgNPs. Note: TEM shows dry-state core diameter; DLS data corresponds to hydrodynamic diameter including hydrated organic surface coating.

A solution of EGCG (1 mM), pre-adjusted to pH 11.5, was mixed with AgNO_3_ (1 mM) and subjected to sustained vortices for 5 min at 25 °C. The resulting stable nano-silver solution is referred to as EAgNPs (Figure 1A). As illustrated in Figure 1B, the formation of AgNPs was confirmed by the presence of a UV–vis absorption band, which peaked at 410 nm [31]. HPLC analysis indicated that EGCG was fully oxidized in the EAgNPs solution prepared with 1 mM EGCG and 1 mM AgNO_3_ (Figure S1). DLS gives a hydrodynamic diameter (including a metallic core plus hydrated organic coating) of approximately 7.6 nm (Figure 1C); TEM reflects the dry-state core size of nanoparticles, with an average diameter of 5.00 ± 1.13 nm (Figure 1D,E). The thermal colloidal stability of EAgNPs in pure aqueous dispersion was further evaluated after incubation at 90 °C for 72 h. Only minor shifts in hydrodynamic size, PDI and zeta potential were observed (Figure S2), demonstrating that the EGCG-derived organic coating confers intrinsic thermal anti-aggregation capability to EAgNPs under harsh aqueous conditions. Furthermore, the colloidal stability of EAgNPs was assessed in serum-containing RPMI-1640 cell-culture medium. After 24 h incubation at 37 °C, moderate increases in hydrodynamic diameter were observed for 30 μM and 100 μM EAgNPs (Table S1), which can be attributed to interactions between serum components and nanoparticle surfaces [39]. Nevertheless, the overall PDI and zeta potential underwent only limited alterations, indicating that no particle agglomeration occurred and EAgNPs retained acceptable colloidal stability under culture-relevant conditions.

To identify the functional groups on the surface of nanoparticles, EAgNPs were analyzed using FTIR spectroscopy (Figure 1F). The FTIR spectrum reveals peaks at 1380 cm^−1^, corresponding to C–O stretching of esters at 1630 cm^−1^, attributed to the stretching of hydrogen-bonded C=O and OH, and at 3440 cm^−1^, associated with the stretching of phenolic –OH groups. XRD analysis confirmed the face-centered cubic crystal structure of the synthesized nanoparticles. The XRD patterns of EAgNPs (Figure 1G) exhibited several diffraction peaks at 2θ = 38.1°, 44.3°, 64.4°, 77.4°, and 81.5°, which correspond to the (111), (200), (220), (311), and (222) diffraction planes, respectively (JCPDS No. 87-0717). To analyze the chemical composition of EAgNPs, XPS was performed. The peaks for C 1s, N 1s, O 1s, Na 1s, and Ag 3d were distinctly observed (Figure 1H). The high-resolution XPS spectrum for Ag 3d in Figure 1I displays two peaks at 368.02 eV (Ag 3d5/2) and 374.02 eV (Ag 3d3/2). The energy separation of 6 eV between these two peaks indicates the metallic state of EAgNPs [40].

### 3.2. Anticancer Activity of the EAgNPs in Tca8113 Cells

**Figure 2:**
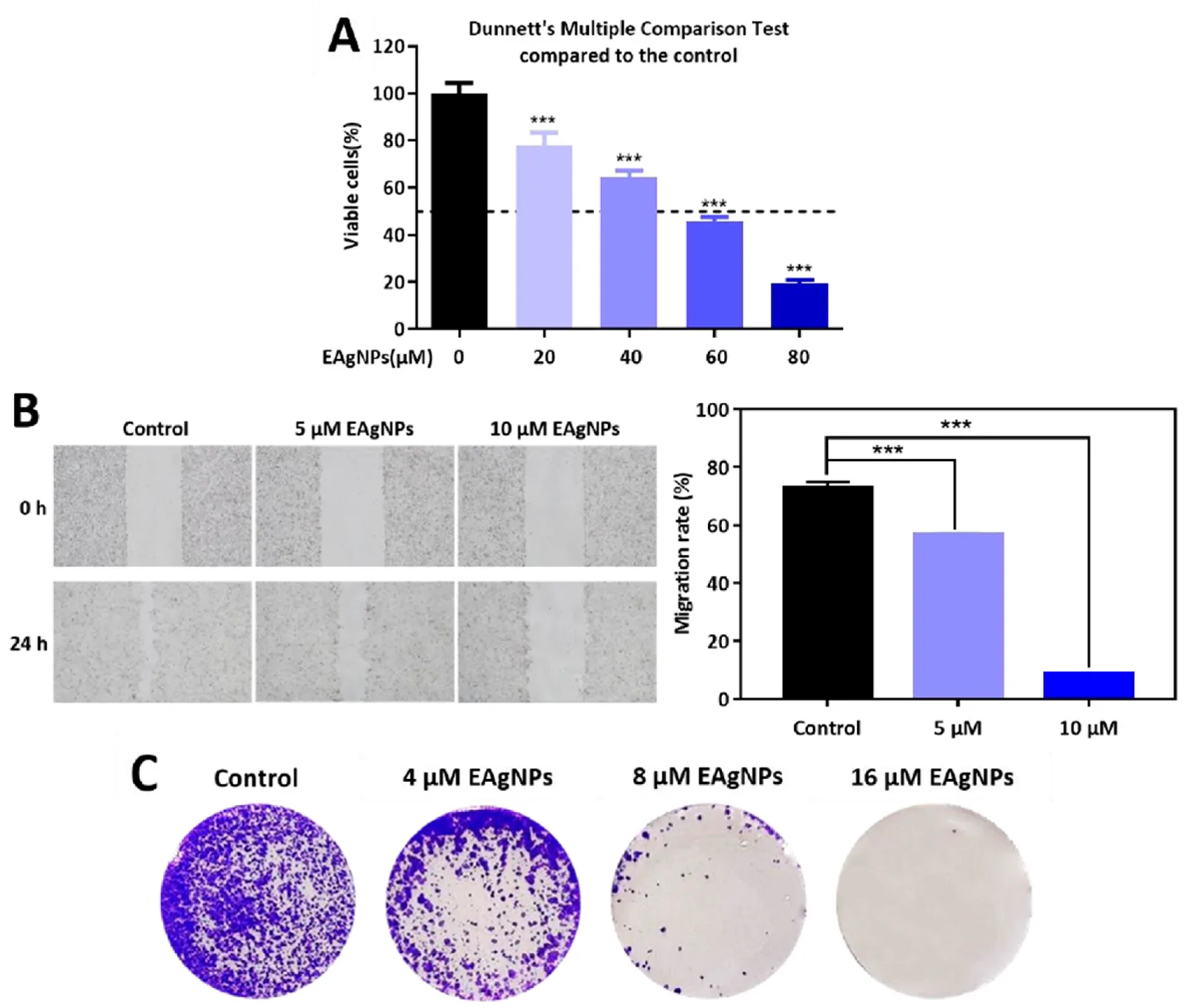
Anticancer activity of EAgNPs. (**A**) Effect of EAgNPs on cell viability in Tca8113 cells. (**B**) Influence of EAgNPs on cell migration in Tca8113 cells. (**C**) Influence of EAgNPs on colony formation in Tca8113 cells. Data are presented as the mean ± SEM (*n* = 6 in **A**,**C** or 3 in **B**). *** *p* < 0.001 vs. control.

To evaluate the effect of EAgNPs on Tca8113 cells, we treated the cells with EAgNPs at concentrations ranging from 20 to 80 µM, based on the AgNO_3_ concentration, and performed a cell viability assay (MTT) after 24 h (Figure 2A). The results indicated that EAgNPs decreased the viability of Tca8113 cells in a dose-dependent manner, yielding an IC_50_ of 47.40 ± 1.68 μM. Furthermore, EAgNPs at concentrations of 5 to 10 µM demonstrated significant efficacy in inhibiting the migration of Tca8113 cells (Figure 2B). Colony formation assays showed that EAgNPs at concentrations of 8 or 16 μM nearly completely suppressed the colony formation of Tca8113 cells (Figure 2C).

**Figure 3:**
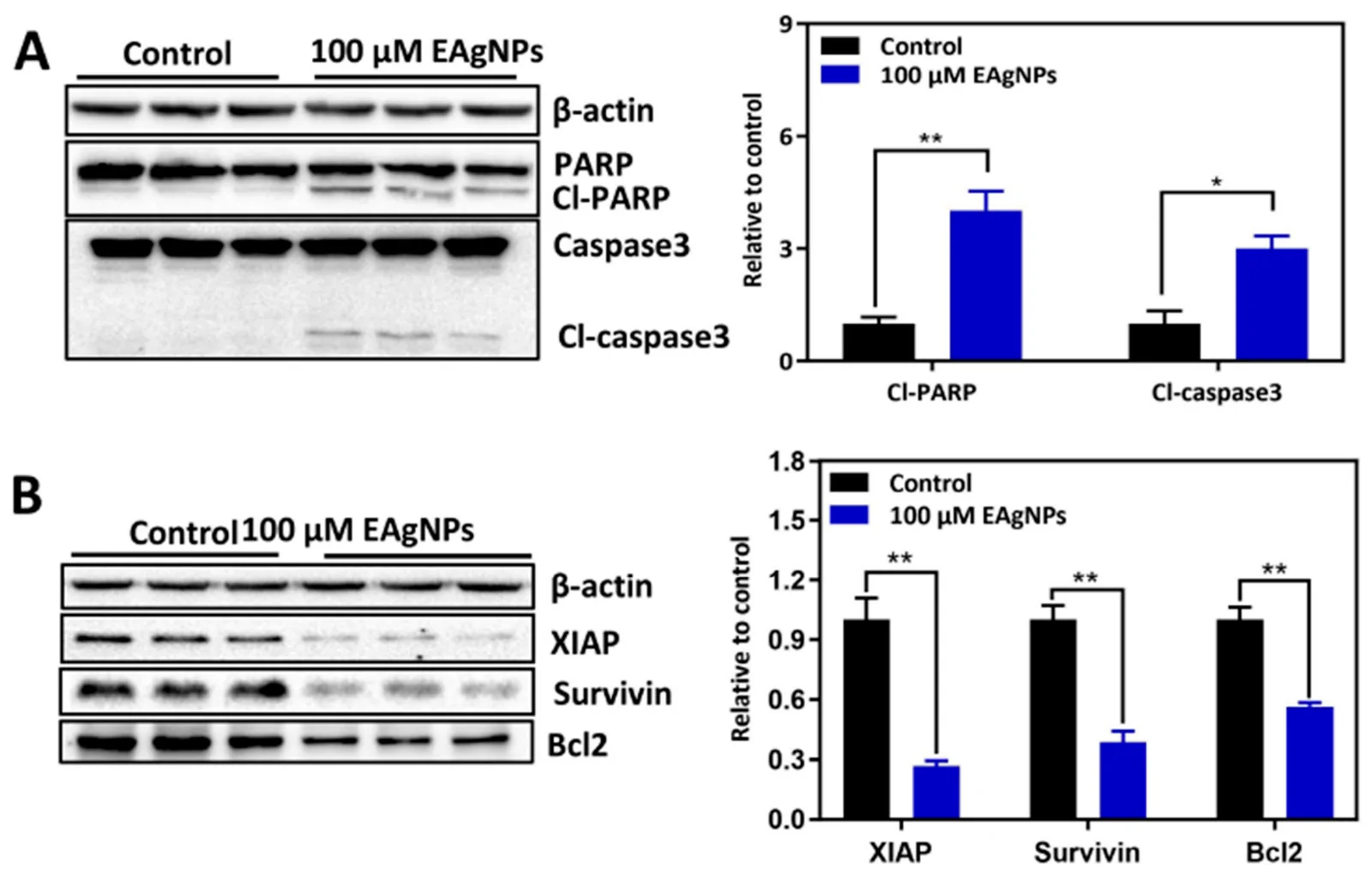
Apoptosis-associated proteins affected by EAgNPs in Tca8113 cells. (**A**) Apoptosis proteins. (**B**) Apoptosis-regulated protein XIAP and pro-survival protein survivin and Bcl-2. Data are presented as mean ± SEM (*n* = 3). Compared to the control, * *p* < 0.05 and ** *p* < 0.01.

We selected a dose of EAgNPs capable of eliminating nearly all cancer cells to investigate their anticancer mechanism. Based on the cell viability assay, we chose 100 μM EAgNPs to assess their impact on the expression of apoptosis-related proteins in Tca8113 cells. At this concentration, EAgNPs upregulated the apoptosis marker proteins Cl-PARP and Cl-caspase3 (Figure 3A), while downregulating the pro-survival proteins XIAP and survivin (Figure 3B). These results indicate that high doses of EAgNPs induce a pronounced apoptotic response.

### 3.3. Effects of a High-Dose EAgNPs on Selenoenzymes, Drug Resistance-Associated Proteins, Antioxidant Enzymes, and GSH Levels in Tca8113 Cells

**Figure 4:**
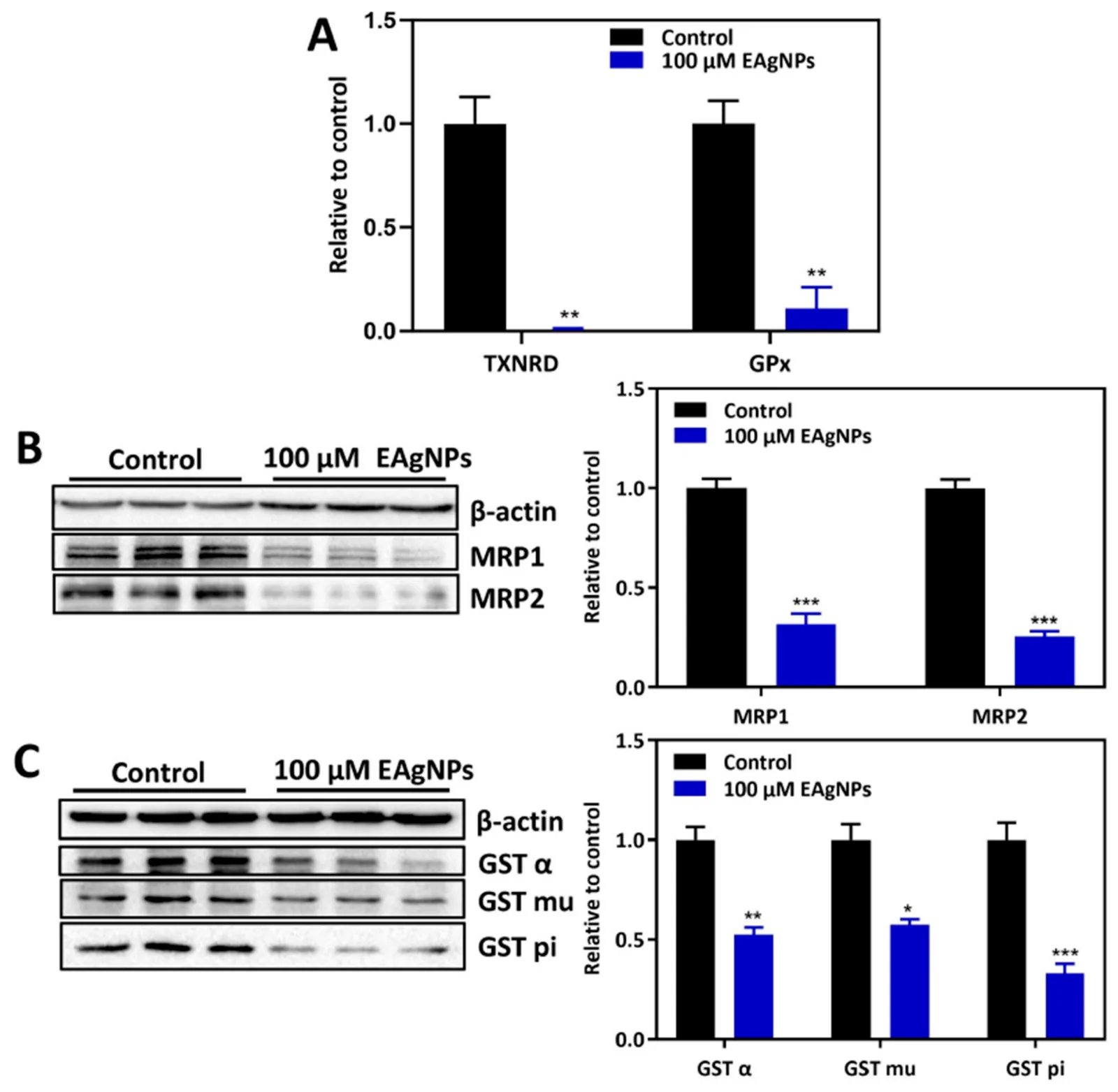
Selenoprotein system and multidrug resistance (MDR)-associated proteins affected by EAgNPs in Tca8113 cells. (**A**) Enzyme activity of TXNRD and GPx. (**B**) Multidrug resistance proteins. (**C**) GST proteins. Data are presented as mean ± SEM (*n* = 3). Compared to the control, * *p* < 0.05, ** *p* < 0.01 and *** *p* < 0.001.

We tested our hypothesis regarding the role of selenoenzyme downregulation in EAgNPs-induced apoptosis. Of note, the present data only show a correlation between selenoenzyme inhibition and apoptosis and cannot confirm a direct causal relationship. Following a 24 h treatment with EAgNPs (100 μM), we observed a significant reduction in the activities of the selenoenzymes TXNRD and GPx in Tca8113 cells (Figure 4A). Additionally, Western blot analysis revealed that EAgNPs treatment led to a downregulation of drug resistance molecules, including MRP1, MRP2, Bcl2, and various isoforms of GST, specifically GSTα, GSTmu, and GSTpi (Figure 4B,C).

**Figure 5:**
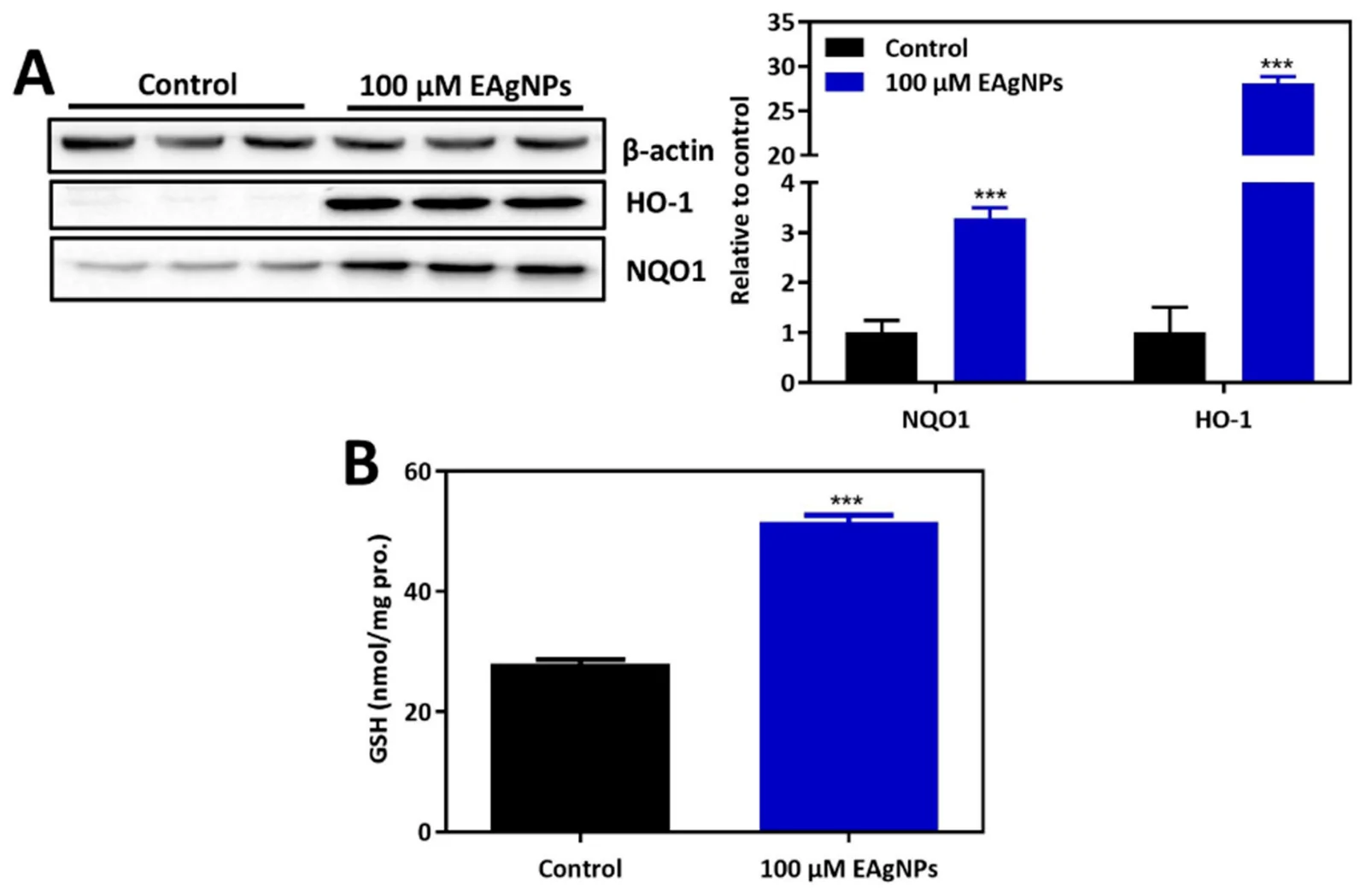
Antioxidant system affected by EAgNPs in Tca8113 cells. (**A**) HO-1 and NQO1 proteins. (**B**) GSH level. Data are presented as mean ± SEM (*n* = 3). Compared to the control, *** *p* < 0.001.

A noteworthy discovery was the significant upregulation of HO-1 and NQO1, crucial downstream effectors of Nrf2, following treatment with 100 μM EAgNPs in Tca8113 cells (Figure 5A). Furthermore, the treatment with 100 μM EAgNPs resulted in an elevation of intracellular GSH levels (Figure 5B). GSH, a key component in cellular antioxidation systems, plays a vital role in eliminating ROS, preventing oxidative stress, and enhancing the drug resistance capabilities of cancer cells [41,42]. However, in this experiment, the increase in Nrf2-associated antioxidant proteins and GSH levels was insufficient to protect the cancer cells from apoptosis induced by the overwhelming oxidative stress and the downregulation of drug resistance molecules and selenoenzymes caused by EAgNPs.

### 3.4. Effects of the Combination of EAgNPs and BSO on Apoptosis and the Antioxidant System in Tca8113 Cells

**Figure 6:**
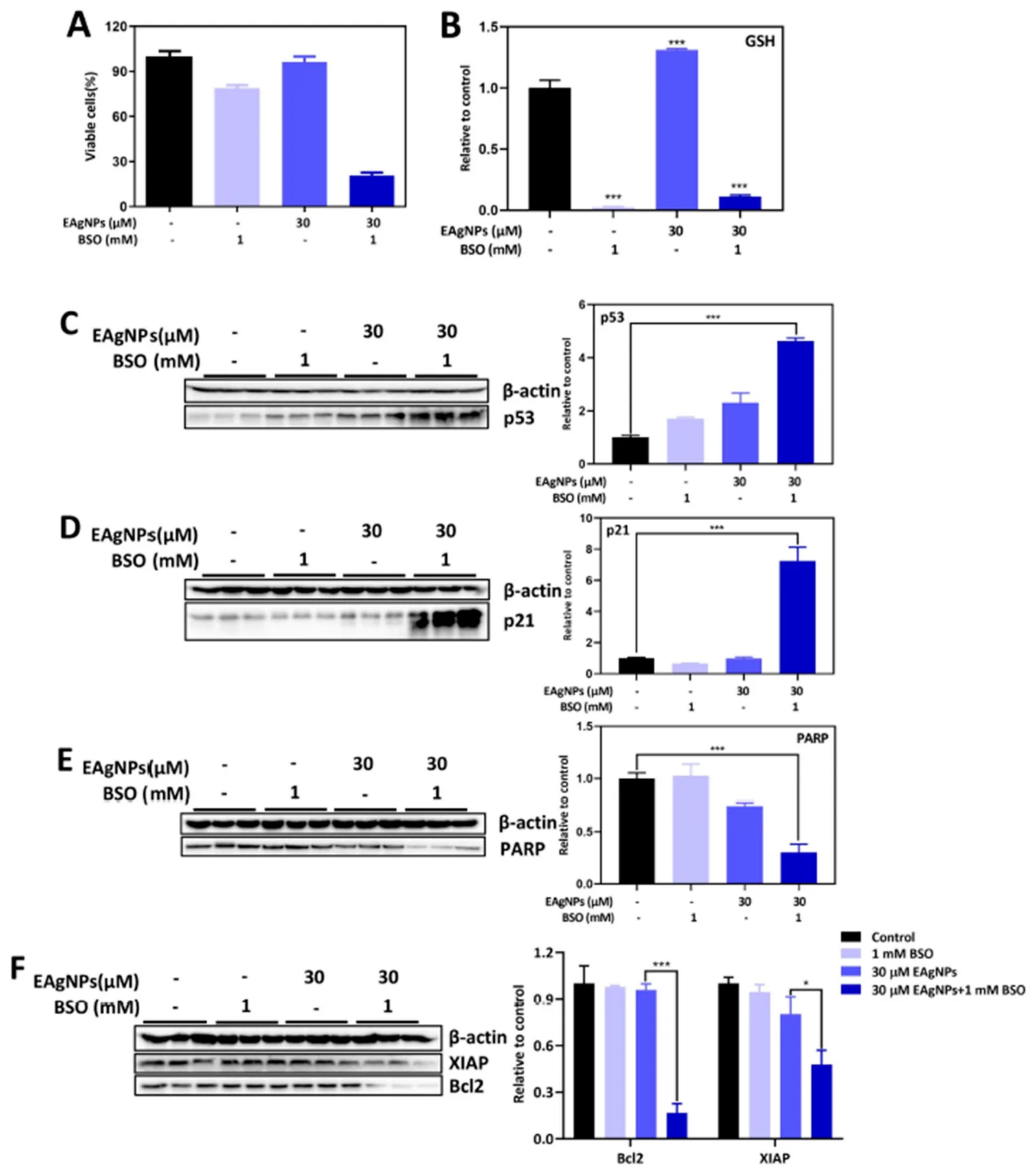
Anticancer activity of EAgNPs and its combination with BSO in Tca8113 cells. (**A**) Effects on cell viability in Tca8113 cells. (**B**) GSH level. (**C**) p53 protein. (**D**) p21 protein. (**E**) PARP protein. (**F**) Pro-survival proteins. Data are presented as mean ± SEM (*n* = 3). Compared to the control, * *p* < 0.05 and *** *p* < 0.001.

Previous studies have demonstrated that BSO can significantly decrease GSH levels [43]. We hypothesized that BSO could enhance the anticancer effects of EAgNPs in Tca8113 cells. To investigate the combined effects, BSO (1 mM) was administered in conjunction with a low dose of EAgNPs (30 µM) in Tca8113 cells (Figure 6A). The combined treatment resulted in cytotoxicity, whereas AgNPs (30 µM) or BSO alone did not exhibit such an effect. As anticipated, the combination led to a decrease in intracellular GSH levels (Figure 6B). Furthermore, the combination increased the levels of apoptosis-related proteins p53 and p21 (Figure 6C,D), while decreasing the levels of pro-survival-related proteins PARP, XIAP, and Bcl2 (Figure 6E,F). Notably, neither EAgNPs (30 µM) nor BSO alone demonstrated a significant effect on the expression of these proteins (Figure 6C,F).

**Figure 7:**
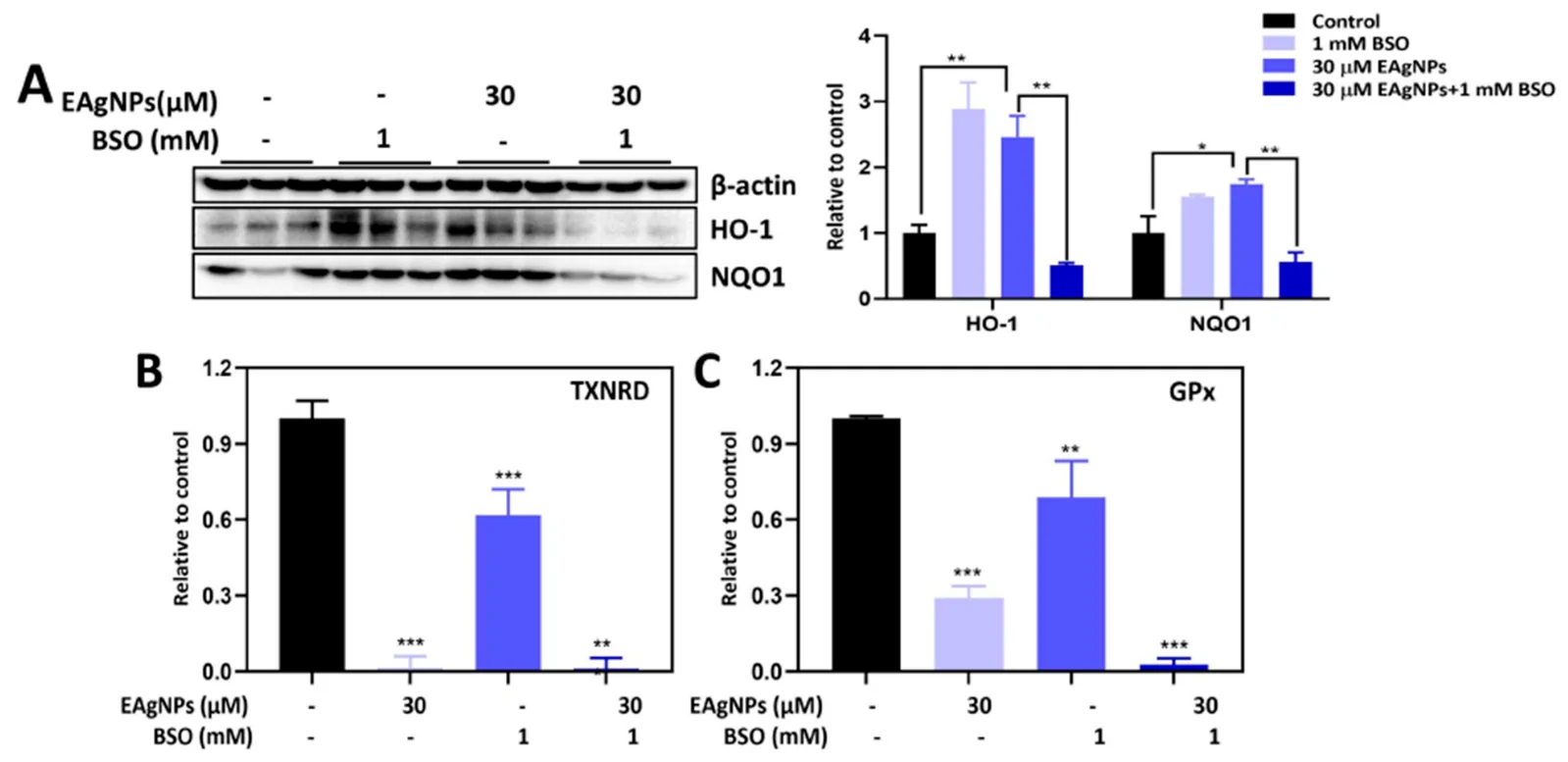
Influence on the antioxidant system by EAgNPs and their combination with BSO in Tca8113 cells. (**A**) HO-1 and NQO1 proteins. (**B**) TXNRDactivity. (**C**) GPx activity. Data are presented as mean ± SEM (*n* = 3). Compared to the control, * *p* < 0.05, ** *p* < 0.01 and *** *p* < 0.001.

The increased cytotoxicity of EAgNPs in the presence of BSO was associated with the downregulation of the Nrf2-associated antioxidant proteins. Our findings indicate that treatment with EAgNPs (30 µM) or BSO alone resulted in an upregulation of NQO1 and HO-1 in Tca8113 cells (Figure 7A). However, the combined treatment of EAgNPs and BSO led to a significant downregulation of both NQO1 and HO-1 (Figure 7A). Notably, this combination treatment almost completely abolished the activities of TXNRD and GPx (these changes were accompanied by enhanced apoptotic phenotype, though causal linkage cannot be concluded from current data) (Figure 7B,C). These results suggest that the combined treatment effectively downregulates the cellular antioxidant defense system.

### 3.5. Effects of the Combination of EAgNPs and BSO on Resistance-Associated Proteins in Tca8113 Cells

**Figure 8:**
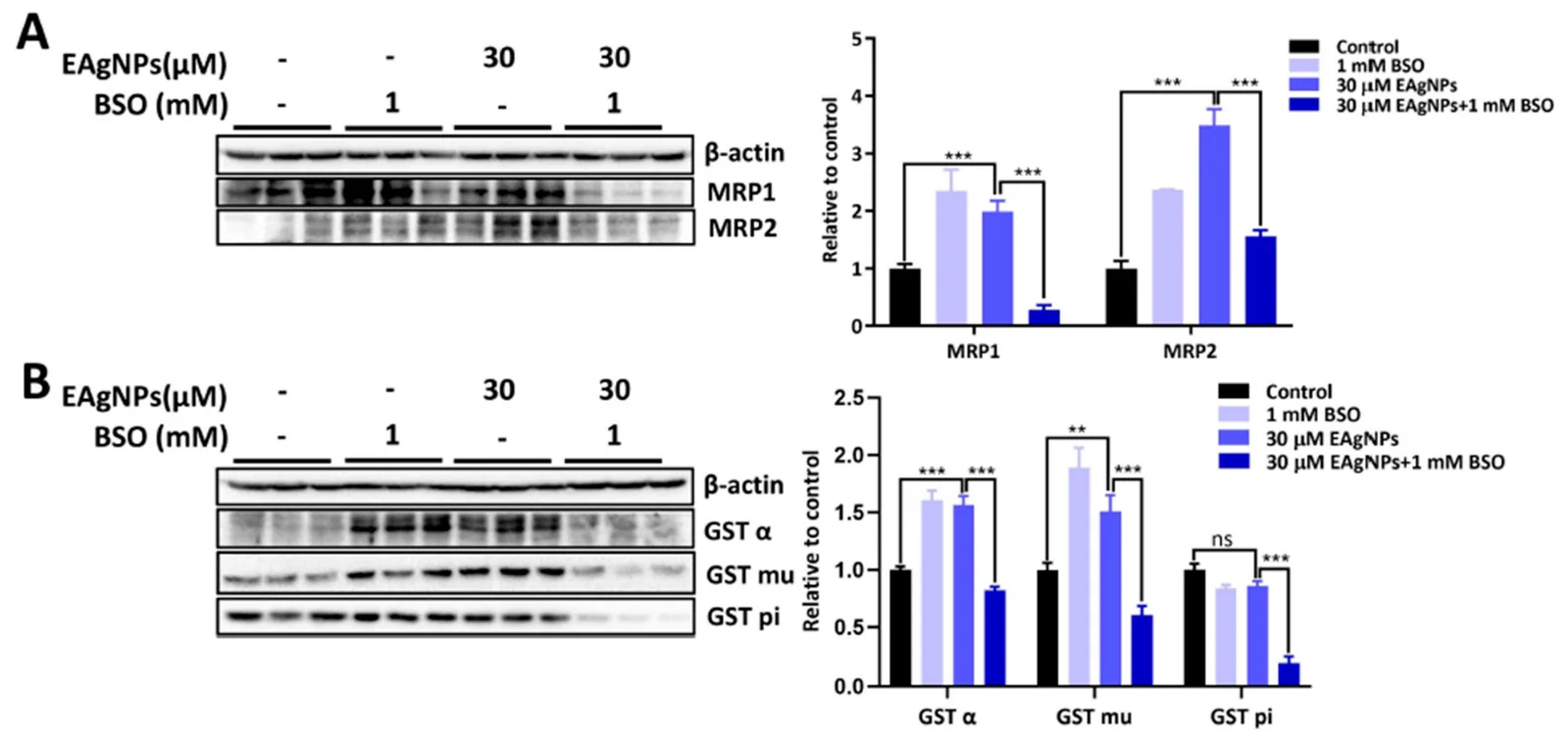
Multidrug Resistance (MDR)-associated proteins affected by EAgNPs and their combination with BSO in Tca8113 cells. (**A**) Multidrug resistance proteins. (**B**) GST proteins. Data are presented as mean ± SEM (*n* = 3). Compared to the control, ns, not significant, *p* > 0.05, ** *p* < 0.01 and *** *p* < 0.001.

Upon investigating the factors contributing to drug resistance, we found that treatment with EAgNPs (30 µM) or BSO significantly upregulated the expression of MRP1, MRP2, and GST isoforms (GSTα and GSTmu) in Tca8113 cells (Figure 8A,B). However, the combination of EAgNPs with BSO resulted in a significant downregulation of these proteins, as well as the GST isoform GSTpi (Figure 8A,B). These findings suggest that low doses of EAgNPs in conjunction with BSO can effectively downregulate drug-resistant proteins, leading to decreased cellular resistance and increased cytotoxicity.

## 4. Discussion

AgNPs are well-documented for their potent tumoricidal activity, primarily through the induction of intracellular ROS overaccumulation [8,9,10]. However, their clinical application is impeded by two significant challenges: severe systemic toxicity at elevated doses and the development of adaptive antioxidant resistance in tumor cells following low-dose exposure [44]. In this study, we biosynthesized EAgNPs utilizing a green bioreduction method and systematically characterized the effects of low and high doses of EAgNPs on selenoenzymes, GSH content, Nrf2-associated antioxidant proteins, and the multidrug resistance network in human tongue squamous cell carcinoma Tca8113 cells. Consistent with our phenotypic data, EAgNPs inhibited cell viability, migration, and colony formation of Tca8113 cells in a concentration-dependent manner, while also suppressing the catalytic activities of two key selenoenzymes, TXNRD and GPx, across all tested concentrations. Importantly, we demonstrated that the glutathione synthesis inhibitor BSO can negate tumor adaptive cytoprotection and enhance pro-apoptotic anti-tumor efficacy when combined with low-dose EAgNPs.

AgNPs have long been recognized as an anti-tumor nanoplatform, but their clinical translation has been severely restricted by systemic toxicities triggered by the high effective doses required to produce satisfactory tumoricidal effects [44]. The present study synthesized and characterized EGCG-derived EAgNPs, and further clarified their distinct dose-dependent anticancer action mechanisms in human tongue squamous carcinoma Tca8113 cells, as well as the enhanced therapeutic effect when combined with the glutathione synthesis inhibitor BSO. These findings fill critical gaps in our understanding of the molecular interactions between AgNPs and the intrinsic pro-survival cascades of cancer cells, and provide a novel, low-toxicity intervention strategy for localized oral cancer treatment.

Our functional characterization results first confirm that EAgNPs exert potent multi-dimensional anticancer activities in Tca8113 cells, manifested as dose-dependent suppression of cell viability, notable inhibition of cell migration even at low concentrations of 5 to 10 μM (Figure 2B), and near-complete abrogation of colony formation at 8 or 16 μM (Figure 2C). These observations are consistent with the well-documented tumoricidal properties of silver nanoparticles [8,9,10], but further extend the current knowledge by identifying a distinct dual-mode dose–response pattern that has not been fully reported in previous AgNPs anti-tumor studies. The 100 μM lethal dose of EAgNPs is sufficient to trigger a robust apoptotic response, evidenced by the upregulation of apoptosis marker proteins Cl-PARP and Cl-caspase3, accompanied by significant downregulation of key pro-survival proteins including XIAP, survivin and Bcl-2 (Figure 3). Mechanistically, this high concentration potently suppresses the activity and expression of core antioxidant selenoenzymes TXNRD and GPx, and simultaneously downregulates multiple key multidrug resistance proteins, including MRP1, MRP2 and various GST isoforms (GSTα, GSTmu, GSTpi) (Figure 4). Notably, even under this lethal treatment condition, Tca8113 cells still initiate partial activation of the Nrf2-associated adaptive defense response, represented by a 2-fold elevation of intracellular GSH levels and 25-fold upregulation of the downstream antioxidant protein HO-1 (Figure 5). However, this compensatory protective response is insufficient to counteract the overwhelming oxidative stress induced by high-dose EAgNPs, as the global impairment of protein biosynthesis and the widespread downregulation of drug resistance and antioxidant defense molecules far exceed the neutralization capacity of the induced antioxidant mediators, ultimately leading to irreversible apoptotic cell death. It should be noted that our current data only show correlative changes between selenoenzyme suppression and apoptosis and cannot establish direct causal evidence. This result clarifies that the intrinsic anticancer potency of high-dose EAgNPs can override partial cytoprotective responses, but it also exposes the fact that the full activation of this tumoricidal effect requires a very high AgNP concentration, which inevitably brings about non-negligible systemic toxicity risks for in vivo application.

By contrast, the 30 μM low-dose EAgNPs that failed to produce salient acute cytotoxicity in our experiments did not represent a biologically ineffective concentration. Instead, they induced a comprehensive integrated cancer cell defense network that promotes adaptive treatment resistance. Low-dose EAgNPs moderately inhibited the activity of the selenoenzyme GPx, but this partial disruption of redox homeostasis triggered full-scale compensatory activation of downstream survival cascades: significant accumulation of intracellular GSH, notable upregulation of Nrf2-associated antioxidant proteins HO-1 and NQO1, and concurrent elevation of the full panel of multidrug resistance proteins, including MRP1, MRP2 and all tested GST isoforms (Figure 6B, Figure 7A and Figure 8). This cytoprotective network effectively neutralizes the moderate oxidative stress induced by low-dose EAgNPs, allowing tumor cells to evade the cytotoxic effect of the nanoparticles and develop intrinsic adaptive resistance [17,45,46,47], which explains why sublethal doses of AgNPs often fail to produce satisfactory therapeutic outcomes in long-term anti-tumor interventions. This discovery also verifies our initial scientific hypothesis that AgNPs-mediated TXNRD inhibition can induce GSH and Nrf2-associated adaptive cytoprotection that counteracts the tumoricidal effect of AgNPs, a mechanism that has been largely overlooked in previous AgNPs anti-tumor research.

Based on the elucidated adaptive resistance mechanism induced by low-dose EAgNPs, our combination strategy using the GSH synthesis inhibitor BSO achieved a highly desirable enhanced therapeutic effect. The co-treatment of 30 μM EAgNPs and BSO not only further enhanced the inhibition of GPx, but also comprehensively abrogated all the inducible anti-apoptotic responses triggered by low-dose EAgNPs monotherapy. BSO effectively depleted the elevated intracellular GSH levels, blocked the compensatory activation of theNrf2-associated antioxidant proteins represented by HO-1 and NQO1 upregulation, and reversed the adaptive upregulation of all multidrug resistance proteins including MRP1, MRP2 and all GST isoforms (Figure 6B, Figure 7A and Figure 8). Again, the changes in selenoenzyme activities were accompanied by enhanced apoptosis, without definite causal proof. This multi-target disruption of the entire cancer cell cytoprotective system subsequently activated the p53/p21 apoptotic axis, significantly upregulated the tumor suppressor proteins p53 and p21, downregulated pro-survival proteins XIAP and Bcl2, and induced robust apoptotic cell death that was not observed with EAgNPs or BSO single-agent treatment (Figure 7C–F). This combinatorial intervention successfully lowered the therapeutic dose threshold of EAgNPs to a sublethal concentration that would otherwise produce no significant cytotoxicity, effectively avoiding the high systemic toxicity risk associated with high-dose nanosilver application while retaining potent tumoricidal activity.

Notably, this combinatorial strategy has prominent translational advantages for oral tongue squamous cell carcinoma treatment. As a localized malignant tumor located in the oral cavity, oral tongue cancer is particularly suitable for topical administration of the nanotherapeutic system, which can achieve high local drug accumulation at the tumor site without requiring high systemic circulating concentrations of AgNPs [48,49]. By disabling the adaptive resistance defense network of cancer cells, the EAgNPs-BSO combination achieves strongly enhanced apoptosis induction at a relatively low EAgNPs dose, which minimizes the potential off-target toxicity of silver nanoparticles to adjacent normal oral tissues and distal organs. Future in vivo investigations in orthotopic tongue cancer animal models are warranted to further verify the in vivo anticancer efficacy, biosafety profile, and tissue distribution characteristics of this combinatorial formulation, to accelerate its translation into clinical applications for localized oral cancer intervention.

Several limitations should be acknowledged in the present work: (1) All cellular experiments were only performed in the Tca8113 tongue cancer cell line, and the observed molecular responses may not represent all subtypes of oral squamous cell carcinoma; (2) No parallel experiments on normal oral keratinocytes were conducted to evaluate tumor-selective cytotoxicity; (3) Cell experiments were performed in medium containing 10% fetal bovine serum. Serum-borne components may modify the physicochemical properties and biological behaviors of nanoparticles, and we have not performed parallel assays under serum-free conditions to independently assess such influences; (4) Although we have assessed the colloidal properties of EAgNPs in serum-containing medium by DLS and sedimentation observation, these measurements cannot fully capture all dynamic physicochemical alterations occurring under long-term cell-culture conditions.

In summary, this study not only clarifies the distinct dose-dependent anticancer modes of biogenic EAgNPs and the underlying adaptive resistance mechanisms in tongue cancer cells, but also proposes a novel combinatorial strategy to target the multi-layered cancer cell cytoprotective system, providing a low-toxicity, translationally feasible new approach for oral cancer treatment.

## 5. Conclusions

In this study, we successfully biosynthesized stable, well-dispersed EAgNPs (4–7.6 nm) and systematically elucidated their dose-dependent antitumor mechanisms in human tongue cancer Tca8113 cells. We identified a distinct dual-mode action: a lethal dose (100 μM) induces robust apoptosis by overwhelming cellular defenses, despite a compensatory Nrf2-associated response (evidenced by a 2-fold increase in GSH and 25-fold upregulation of HO-1). Conversely, a sublethal dose (30 μM) triggers a comprehensive cytoprotective network, characterized by GSH accumulation and upregulated drug resistance proteins. Crucially, we demonstrated that co-treatment with the glutathione synthesis inhibitor BSO effectively abrogates this adaptive resistance. This combinatorial strategy disrupts both selenoenzyme and Nrf2-associated antioxidant proteins while reversing multidrug resistance, thereby inducing enhanced apoptosis via the p53/p21 axis. By significantly lowering the therapeutic threshold, this approach presents a promising, low-toxicity topical strategy for oral cancer treatment, meriting further in vivo validation.

## Data Availability

The original contributions presented in this study are included in the article/Supplementary Material. Further inquiries can be directed to the corresponding author(s).

## Supplementary Materials

The following supporting information can be downloaded at: https://www.mdpi.com/article/10.3390/molecules31193536/s1, Figure S1: Chromatogram and standard curve of EGCG detected by HPLC. (A) Chromatogram of EGCG assessed by HPLC. (B) Chromatogram of EAgNPs assessed by HPLC. Figure S2: Thermal stability of EAgNPs after treatment at 90 °C for 72 h characterized by DLS. EAgNPs samples stored frozen in a refrigerator were thawed and divided into two groups: the control group was incubated at room temperature for 72 h, and the experimental group was subjected to thermal treatment at 90 °C for 72 h. (A) Particle size distribution, (B) Zeta potential, and (C) Polydispersity index (PDI) of EAgNPs in different groups. All data are presented as mean ± SEM (*n* = 6). Table S1: Influence of RPMI-1640 medium containing 10% FBS incubation at 37 °C for 24 h on the stability of EAgNPs characterized by DLS.

## Abbreviations

AgNPs, silver nanoparticles; BSO, L-Buthionine-sulfoximine; DLS: dynamic light scattering; EAgNPs, EGCG-derived silver nanoparticles; EGCG, (−)-epigallocatechin-3-gallate; GPx, Glutathione peroxidase; GSH, glutathione; HPLC: high performance liquid chromatography; GST, glutathione transferase; MDR, multiple drug resistance; MRP, multidrug resistance-associated protein; MTT: 3-(4,5-Dimethyl-2-thiazolyl)-2,5-diphenyltetrazolium bromide; Nrf2, nuclear factor E2-related factor 2; ROS, reactive oxygen species; Tca8113, human tongue squamous cell line; TEM: transmission electron microscope; TXN, thioredoxin; TXNRD, thioredoxin reductase; XPS: X-ray photoelectron spectroscopy; XRD: X-ray diffraction.
