# Supplementary material for: Responses of Anti-Apoptotic Molecules to EAgNPs and EAgNPs Plus L-Buthionine-Sulfoximine in Human Tongue Cancer Cells
Source: Molecules. 2026 Oct 4;31(19):3536. doi: 10.3390/molecules31193536 (PMC13667187; doi:10.3390/molecules31193536)
Supplement: Supplementary file 1 [file molecules-31-03536-s001.zip › molecules-4501481-supplementary.pdf]

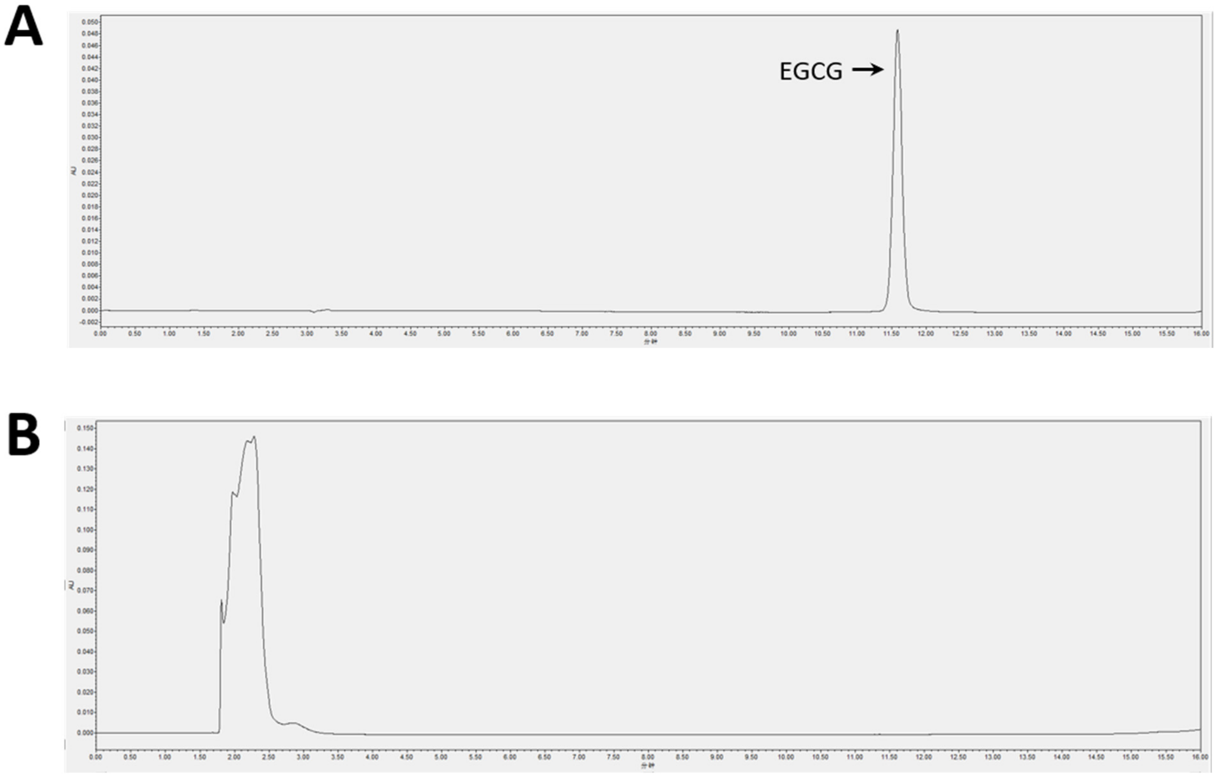

Figure S1 Chromatogram and standard curve of EGCG detected by HPLC. (A) Chromatogram of EGCG assessed by HPLC. (B) Chromatogram of EAgNPs assessed by HPLC.

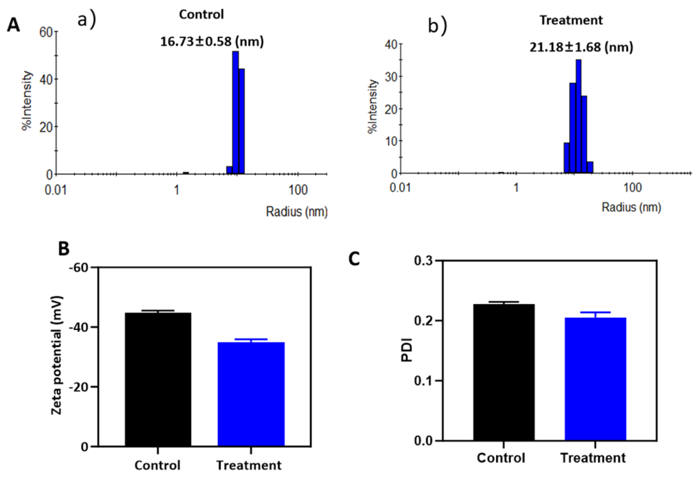

Figure S2. Thermal stability of EAgNPs after treatment at 90 °C for 72 h characterized by DLS.

EAgNPs samples stored frozen in a refrigerator were thawed and divided into two groups: the control group was incubated at room temperature for 72 h, and the experimental group was subjected to thermal treatment at 90 °C for 72 h. (A) Particle size distribution, (B) Zeta potential, and (C) Polydispersity index (PDI) of EAgNPs in different groups. All data are presented as mean ± SEM (n=6).

Table S1. Influence of RPMI-1640 medium containing 10 % FBS incubation at 37℃ for 24 h on the stability of EAgNPs characterized by DLS.

Measurement Items	30 μM EAgNPs	100 μM EAgNPs
Diameter (nm)	18.75±1.21	19.63±2.23
PDI	0.31±0.02	0.28±0.02
zeta potential (mV)	-36.71± 0.31	-38.71± 0.34

Data are presented as the mean ± SEM (n=6).
